# Absence of Anti-RBD Antibodies in SARS-CoV-2 Infected or Naive Individuals Prior to Vaccination with CoronaVac Leads to Short Protection of Only Four Months Duration

**DOI:** 10.3390/vaccines10050690

**Published:** 2022-04-28

**Authors:** Camille F. de Oliveira, Walter F. F. Neto, Carla P. da Silva, Ana Claudia S. Ribeiro, Lívia C. Martins, Alana W. de Sousa, Maria N. O. Freitas, Jannifer O. Chiang, Franko A. Silva, Eder B. dos Santos, Daniele B. A. Medeiros, Gleiciane S. Pinheiro, Gleiciane F. Brandão, Valéria L. Carvalho, Raimunda S. S. Azevedo, Pedro F. C. Vasconcelos, Igor B. Costa, Iran B. Costa, Mirleide C. dos Santos, Luana S. Soares, Rayssa L. S. Bedran, James L. Ferreira, Alberto A. Amarilla, Naphak Modhiran, Christopher L. D. McMillan, Morgan E. Freney, David A. Muller, Daniel Watterson, Lívia M. N. Casseb, Daniele F. Henriques

**Affiliations:** 1 Department of Arbovirology and Hemorrhagic Fevers, Evandro Chagas Institute, Ananindeua 67030-000, PA, Brazil; camille.oliveeira@gmail.com (C.F.d.O.); walter96neto@gmail.com (W.F.F.N.); carlapinheiro@iec.gov.br (C.P.d.S.); ana-claudia201189@hotmail.com (A.C.S.R.); liviamartins@iec.gov.br (L.C.M.); alanasousa@iec.gov.br (A.W.d.S.); mariafreitas@iec.gov.br (M.N.O.F.); janniferchiang@iec.gov.br (J.O.C.); frankosilva@iec.gov.br (F.A.S.); eder_b_santos@hotmail.com (E.B.d.S.); danielemedeiros@iec.gov.br (D.B.A.M.); gleicianepinheiro@iec.gov.br (G.S.P.); gleicianebrandao@iec.gov.br (G.F.B.); valeriacarvalho@iec.gov.br (V.L.C.); raimundaazevedo@iec.gov.br (R.S.S.A.); liviacasseb@iec.gov.br (L.M.N.C.); 2 Department of Biological and Health Sciences, University of Pará State, Belém 66087-670, PA, Brazil; pedrovasconcelos@iec.gov.br; 3 Department of Virology, Evandro Chagas Institute, Ananindeua 67030-000, PA, Brazil; igorcosta@iec.gov.br (I.B.C.); irancosta@iec.gov.br (I.B.C.); mirleidesantos@iec.gov.br (M.C.d.S.); luanasoares@iec.gov.br (L.S.S.); rayssabedran@iec.gov.br (R.L.S.B.); jamesferreira@iec.gov.br (J.L.F.); 4 School of Chemistry and Molecular Biosciences, The University of Queensland, St Lucia, QLD 4072, Australia; a.amarillaortiz@uq.edu.au (A.A.A.); n.modhiran@uq.edu.au (N.M.); c.mcmillan1@uq.edu.au (C.L.D.M.); m.freney@uq.edu.au (M.E.F.); d.muller4@uq.edu.au (D.A.M.); d.watterson@uq.edu.au (D.W.); 5 Australian Infectious Disease Research Centre, The University of Queensland, St Lucia, QLD 4072, Australia; 6 The Australian Institute for Biotechnology and Nanotechnology, The University of Queensland, St Lucia, QLD 4072, Australia

**Keywords:** COVID-19, SARS-CoV-2, CoronaVac, vaccines, antibodies

## Abstract

The COVID-19 pandemic is the biggest public health threat facing the world today. Multiple vaccines have been approved; however, the emergence of viral variants such as the recent Omicron raises the possibility of booster doses to achieve adequate protection. In Brazil, the CoronaVac (Sinovac, Beijing, China) vaccine was used; however, it is important to assess the immune response to this vaccine over time. This study aimed to monitor the anti-SARS-CoV-2 antibody responses in those immunized with CoronaVac and SARS-CoV-2 infected individuals. Samples were collected between August 2020 and August 2021. Within the vaccinated cohort, some individuals had a history of infection by SARS-CoV-2 prior to immunization, while others did not. We analyzed RBD-specific and neutralizing-antibodies. Anti-RBD antibodies were detected in both cohorts, with a peak between 45–90 days post infection or vaccination, followed by a steady decline over time. In those with a previous history of COVID-19, a higher, longer, more persistent response was observed. This trend was mirrored in the neutralization assays, where infection, followed by immunization, resulted in higher, longer lasting responses which were conditioned on the presence of levels of RBD antibodies right before the vaccination. This supports the necessity of booster doses of CoronaVac in due course to prevent serious disease.

## 1. Introduction

In December 2019, the first cases of coronavirus disease 2019 (COVID-19) were detected in Wuhan, Hubei Province, China. The disease was caused by the novel human coronavirus SARS-CoV-2 [1], which was declared a pandemic in March 2020. SARS-CoV-2 belongs to the order *Nidovirales,* family *Coronaviridae*, and genus *Betacoronavirus* (β-CoV) [2]. Like all coronaviruses, SARS-CoV-2 is a non-segmented, positive sense, single-stranded RNA virus, with a genome length ranging from 29,891 to 29,903 nucleotides. The genome encodes for four structural proteins: nucleocapsid protein (N) enclosed by a lipid envelope in which the three other structural proteins are embedded: spike (S) divided into two functional subunits, S1 which contains the receptor binding domain (RBD) and S2, membrane (M), and envelope (E) [3].

The infection of host cells by SARS-CoV-2 occurs through the binding of the S protein, via the RBD subunit, to the cellular angiotensin-converting enzyme 2 (ACE2) receptor. These ACE2 receptors are present in several human organs, including lungs, kidneys, liver, gastrointestinal tract, and blood vessels, contributing to the high contagious ability of SARS-CoV-2 [4,5]. COVID-19 presents a spectrum of clinical manifestations ranging from mild illness, representing more than 80% of cases, to severe clinical status in approximately 10% of cases [6,7]. Symptoms of infection include fever, dry cough, anosmia, ageusia, and fatigue, usually with pulmonary involvement. Severe cases present with dyspnea, tachypnea, and low oxygen saturation in the blood, which may lead to death due to respiratory or multiple organ failure [6,7].

The development of vaccines for SARS-CoV-2 is a key strategy to reduce the morbidity and mortality of COVID-19. Globally, 344 SARS-CoV-2 vaccines are under development, of which 195 are in the preclinical phase and 149 are in the clinical phase [8]. These vaccines utilize a variety of development platforms, including inactivated vaccines, attenuated virus vaccines, vectored vaccines, messenger RNA-based vaccines, and recombinant subunit or VLP vaccines [9]. Current pandemic figures (15 March 2022) record 6,047,103 deaths, 459,960,370 cases of SARS-CoV-2 infection, and 10,717,451,112 vaccine doses administered worldwide [10].

In Brazil, the CoronaVac vaccine (inactivated vaccine, manufactured by Sinovac Life Sciences Co. Ltd., Beijing, China), was licensed for production in partnership with the Butantan Institute/Sinovac and was widely used after the release for emergency use by the Brazilian Health Regulatory Agency (Anvisa). The Ministry of Health initially prioritized the vaccination of at-risk groups—elderly people with comorbidities and health professionals [11]. Our study aimed to monitor the production and maintenance of anti-SARS-CoV-2 spike RBD protein antibodies in naturally SARS-CoV-2 infected individuals and those immunized with CoronaVac. Furthermore, we tracked the neutralizing antibodies response against the B.1.1.33 variant, the first variant introduced in Brazil, responsible for driving the epidemic in the country [12], and the variant of concern (VOC), Gamma, in naïve individuals with a previous history of COVID-19 who received two doses of CoronaVac.

## 2. Materials and Methods

### 2.1. Ethical Aspects, Study Population, and Kinetics

**Figure 1:**
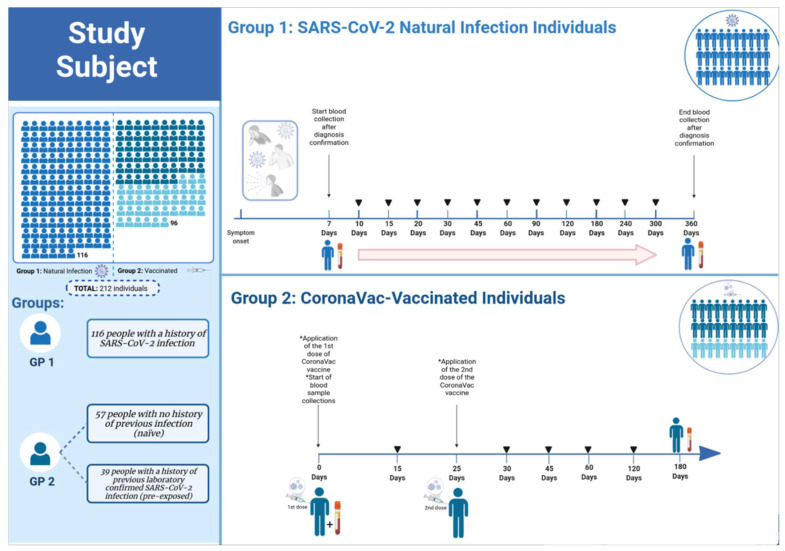
Experimental design of group 1 and group 2. In 116 individuals infected with SARS-CoV-2 belonging to Group 1, serial blood draws were performed alternately between seven days and 12-months post symptom onset. In group 2, 96 individuals vaccinated with CoronaVac were split into two subgroups: 39 with a history of previous laboratory-confirmed SARS-CoV-2 infection and 57 with no history of prior infection (naïve). Blood samples were collected immediately before the first dose, day 0, and serially on days 15, 30, 45, 60, 120, and 180-days post first immunization. All participants received the second dose of the CoronaVac vaccine 25 days after the first dose. GP—Group.

This was a prospective cohort study, conducted between August/2020 and August/2021 at Evandro Chagas Institute, Ananindeua city, state of Pará, Brazil. This study was approved by the Institutional Research Ethics Committee (CAAE 43109021.7.0000.0019) and a written consent form was signed by all volunteers. The cohort was composed of 212 residents in the metropolitan region of Belém, Pará State, Brazil, divided into two groups: those infected with SARS-CoV-2 (Group 1) and those immunized with the CoronaVac vaccine (SINOVAC Biotech Ltd., Beijing, China) (Group 2) (Figure 1). The characteristics of participants are specified in Table 1.

Of these, 116 individuals (belonging to Group 1) were positive for SARS-CoV-2 infection or presented with symptoms consistent with COVID-19. Of the 116 in Group 1, 98 were confirmed to be infected with SARS-CoV-2 by RT-qPCR and the remaining 18 individuals were confirmed by other methodologies (rapid antigen test and ELISA). This group includes asymptomatic individuals, as well as those who manifested mild, moderate, or severe symptoms, but without hospitalizations. In 35 participants, serial blood draws were performed alternately between days 7, 10, 15, 20, 30, 45, 60, 90, and 120-days post symptom onset, while 81 participants had their blood drawn at 6, 8, 10, and 12-months post symptom onset (Figure 1).

In Group 2, 96 individuals vaccinated with CoronaVac were split into two subgroups: 39 with a history of previous laboratory confirmed SARS-CoV-2 infection (pre-exposed within 6–10 months) and 57 with no history of previous infection (naïve). Blood samples were collected immediately before the first dose, day 0 (baseline), and serially on days 15, 30, 45, 60, 120, and 180-days post first immunization (Figure 1). All participants received the second dose of CoronaVac vaccine 25 days after the first dose. Blood samples were collected in vacuum tubes without anticoagulants and were centrifuged at 800× *g* for 10 min to obtain serum, which was stored at −80 °C.

### 2.2. SARS-CoV-2 RBD Specific Enzyme-Linked Immunosorbent Assay (ELISA)

Serum samples were tested for anti-SARS-CoV-2 RBD specific total antibodies by indirect enzyme-linked immunosorbent assay (ELISA) adapted from [13,14]. The receptor-binding domain (RBD) of the spike protein was expressed in ExpiCHO cells and purified as previously described. Briefly, 96-well plates (Thermo Fisher Scientific, Waltham, MA, USA) were coated with 50 µL/well of 2 µg/mL RBD in 0.1 M pH 7.4 buffered saline (PBS) and incubated overnight at 4 °C. The next day, after removal of the coating by three washes with distilled water, 150 µL/well of blocking buffer solution (KPL Milk diluent/Blocking solution concentrate (SeraCare, Milford, MA, USA), diluted in PBS containing 0.5% Tween 20) was added and incubated for 1 h at room temperature. After the blocking step, the plates were washed 3 times by submersion in distilled water and 50 µL/well of diluted serum samples (1:100 in blocking buffer) and controls (positive and negative human sera) were added to the plates and incubated for 1 h at 37 °C. This was followed by three washes with distilled water, before the addition of 50 µL/well of goat anti-human Kappa secondary antibody (Sigma Aldrich, Saint Louis, MO, USA) diluted 1:2000. The plates were incubated for 1 h at 37 °C. Next, unbound secondary antibody was removed by three washes with distilled water and 50 µL/well of HRP-conjugated polyclonal anti-goat antibody conjugated to peroxidase (HRP) (DAKO, Glostrup, Denmark) diluted 1:1000 was added and incubated for 1 h at 37 °C. A final three washes were performed to remove all the unbound polyclonal antibody and then, 50 µL/well of the substrate Tetramethylbezidine -TMB (SeraCare, Milford, MA, USA) was added and incubated in a dark chamber at room temperature (25 °C) for 5 min. The reaction was stopped by the addition of 30 µL/well of stop solution (1M H_2_SO_4_). Optical density (OD) at 450 nm was then read using the LMR-96 microplate reader (Loccus Biotecnologia, Cotia, SP, Brazil). The OD of the blank was subtracted from each sample and the cutoff was determined through the ROC curve. Samples with antibodies with OD ≥ 0.200 were considered positive.

### 2.3. Plaque Reduction Neutralization Test 50% (PRNT50)

Twenty-three vaccinated individuals, 14 with a previous history of SARS-CoV-2 infection and nine without, were chosen randomly. Then, we measured the neutralizing antibodies against B.1.1.33 e Gamma variants before (baseline, D0) and after vaccination (15–180 days). PRNT50 determination was performed as described by [15] with adaptations. Briefly, the samples from CoronaVac-vaccinated individuals and previously selected controls (positive and negative human sera) were heat inactivated (56 °C for 1 h) and two-fold serial dilutions were performed in maintenance medium 199 (M199). Next, diluted serum was incubated with the SARS-CoV-2 variants for 1 h at 37 °C and 5% CO_2_. For the serum-virus complexes, we used an equal amount of virus suspension containing 100 PFU. Then, immunocomplexes were added to 24-well plates containing a confluent monolayer of Vero CCL-81 cells (ATCC, Manassas, VA, USA) and incubated for 1 h at 37 °C and 5% CO_2_. Subsequently, 1.5 mL of the overlay medium (medium 199 containing 1.5% Carboxymethylcellulose and 5% Fetal Bovine Serum-FBS) was added to each well and incubated for 4 days at 37 °C in an atmosphere containing 5% CO_2_. The plates were fixed using 10% formaldehyde for 4 h and then stained with 1% Crystal Violet for 2 h. Plaque counting was performed under an optical microscope (Zeiss, Oberkochen, Germany). After counting the plaques, the neutralizing antibody titers were determined based on the PRNT50, considering positive the sera that reduced the number of plaques by 50% compared to the average of the virus control wells.

### 2.4. Statistical Analyses

All statistical analyses and graphical representation were done using GraphPad Prism software (version 8, San Diego, CA, USA). The Mann–Whitney U test was used to evaluate the statistical difference between the group of immunized participants who had previous natural infection and people with no history of previous natural infection. These groups were stratified by days after vaccination, and the statistical difference was evaluated by multiple comparisons with means of groups using the ANOVA tests. For all analyses, statistical significance was assumed when *p* < 0.05. Mean and standard deviation were calculated to compare the values obtained from optical density (OD) and/or viral neutralization titers. Linear regression and Spearman correlation were used to evaluate the strength and power of correction between obtained OD values and neutralization titers.

## 3. Results

### 3.1. Monitoring of RBD Specific Antibodies Response after SARS-CoV-2 Infection and Post Vaccination with Coronavac Vaccine

**Figure 2:**
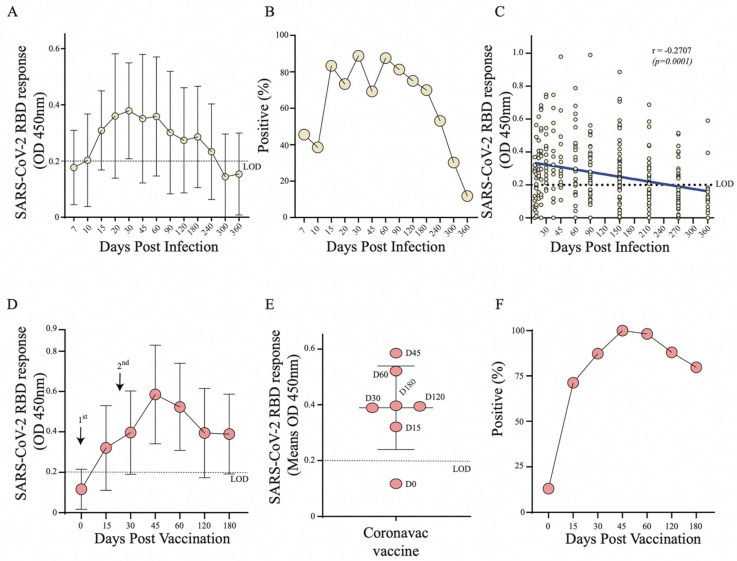
Monitoring of SARS-CoV-2 RBD specific antibodies through ELISA in Group 1 (*n* = 116) and Group 2 (*n* = 96). (**A**) Curve of RBD specific antibodies in SARS-CoV-2 naturally infected individuals (Group 1); (**B**) Percentage of seroconversion in SARS-CoV-2 infected individuals (Group 1); (**C**) Linear regression analysis of anti-RBD antibodies over 12-months from the day of infection by SARS-CoV-2 (Group 1); (**D**) Curve of RBD specific antibodies in CoronaVac vaccinated individuals (Group 2); (**E**) Distribution of OD means post vaccination (Group 2); (**F**) Percentage of seroconversion in CoronaVac vaccinated individuals (Group 2).

To understand the kinetics of RBD specific antibodies and seroconversion acquired in SARS-CoV-2 infection (Group 1) and vaccinated individuals (Group 2), we tested serum samples via an indirect ELISA with a recombinant RBD antigen for SARS-CoV-2 (Ancestral strain that was isolated in Wuhan in 2020). The optimal optical density (OD) values in convalescent serum samples (Group 1, *n* = 116) varied between 0.200 and 0.734 (Figure 2A). In these convalescent participants we observed a progressive increase in OD values and seroconversion from 45.5% at 7 days post infection, to more than 80% seropositivity between 15- and 90-days post infection (Figure 2A,B). Notably, seroconversion decreased after 6 months (70%) with dramatic drop to 50% and 11% seropositivity by 8 and 12 months after infection, respectively (Figure 2B). A linear regression analysis confirmed this progressive reduction of anti-RBD antibodies over the 12-month period from the day of infection (*r* = −0.2707, *p* < 0.0001) (Figure 2C).

In vaccinated individuals (Group 2, *n* = 96), we observed elevated OD values from 15 days after the first dose with a peak between 45- and 60-days post vaccination (Figure 2D,E). Seropositivity reached 100% at 45 after the 1st dose (15 days after the 2nd dose) and declined to 75% by 6 months after the first immunization (Figure 2F). Thus, we found comparable kinetics of RBD specific antibodies responses and seroconversion between convalescent serum and vaccinated individuals.

### 3.2. Profile of RBD Specific Antibody Response in Individuals after Vaccination with Coronavac Vaccine

**Figure 3:**
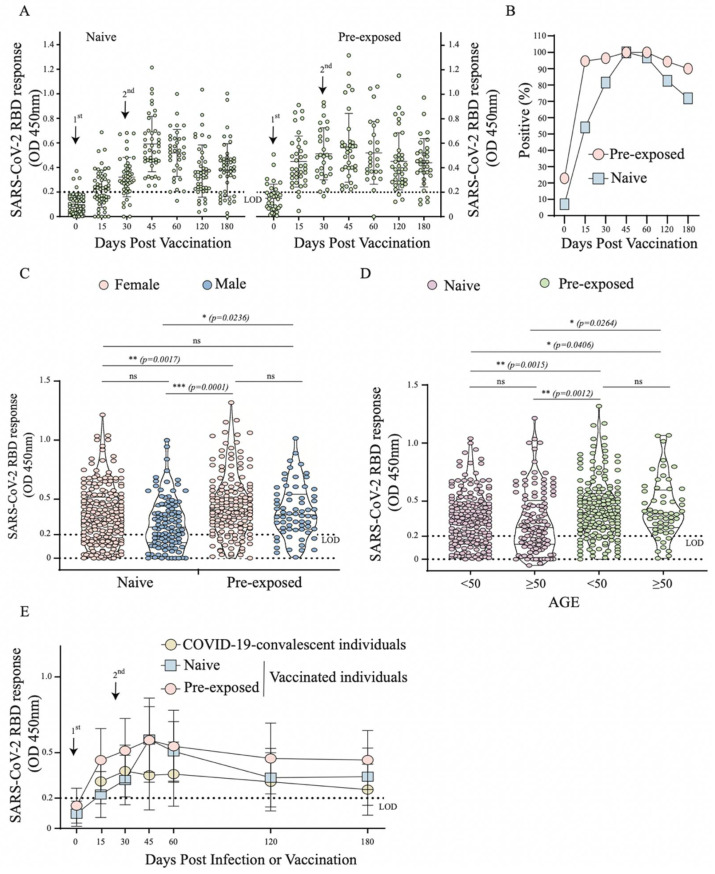
Profile of RBD specific antibody response in individuals after vaccination with CoronaVac vaccine through ELISA in Group 2 (*n* = 96). (**A**) Distribution of OD values per day in Dotplot of the vaccinated naïve and SARS-CoV-2 pre-exposed individuals; (**B**) Percentage de seroconversion of vaccinated naïve and SARS-CoV-2 pre-exposed individuals; (**C**) Comparative analysis of OD values in vaccinated naïve and SARS-CoV-2 pre-exposed individuals by gender (female and male). (**D**) Comparative analysis of OD values in vaccinated naïve and SARS-CoV-2 pre-exposed individuals by age group (<50 and ≥50), (**E**) Curve of RBD specific antibodies in COVID-19-convalescent (Group 1), vaccinated naïve and pre-exposed individuals. *—*p* < 0.05; **—*p* < 0.01; ***—*p* < 0.001; ns—*p* ≥ 0.05.

As 40.6% of participants had a history of SARS-CoV-2 infection before vaccination, from which 22.9% had detectable RBD-specific antibodies by ELISA before receiving the 1st dose (Figure 3B), we decided to further analyse the antibody responses between those vaccinated individuals with or without a history of exposure. As expected, after the 1st dose, vaccinated participants with a history of infection produced a greater RBD specific antibody response, as well as a higher seroconversion rate compared to vaccinated naïve individuals (Figure 3A,B). Interestingly, 15 days after the 2nd dose, both groups seroconverted by 100% with the same level of antibody response (Figure 3B). We also noticed a higher level of antibody response in pre-exposed individuals was independent of gender and age (*p* = 0.0236, *p* = 0.0264, respectively) (Figure 3C,D). In those with a previous history of SARS-CoV-2 infection, vaccination resulted in a higher antibody response that persisted over 6 months post vaccination in a large number of participants (Figure 3B,E). Overall, this suggests that pre-exposure to SARS-CoV-2 infection boosts the antibody response and makes it longer-lasting upon vaccination compared to naïve individuals.

### 3.3. Neutralizing Antibodies Levels in Individuals after Vaccination with Coronavac Vaccine

**Figure 4:**
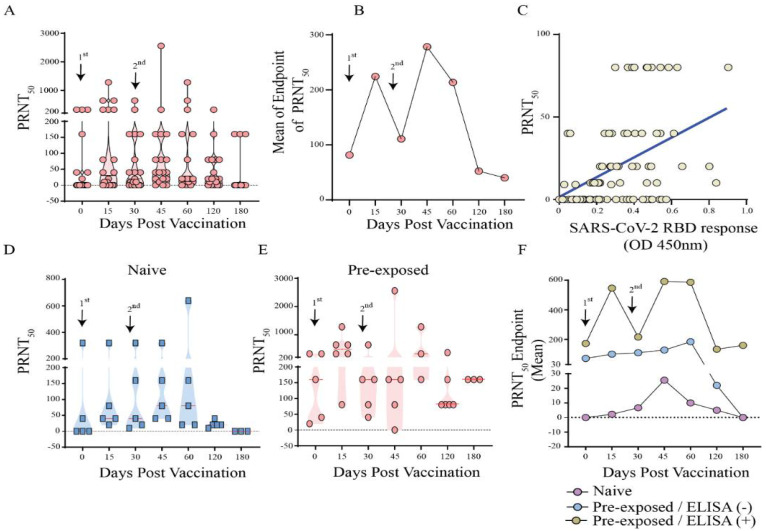
Neutralizing antibodies levels in individuals after vaccination with CoronaVac vaccine (Group 2) against B.1.1.33 variant, the primary strain in Brazil. (**A**) Titer of neutralizing antibodies per day in Dotplot of CoronaVac vaccinated individuals (**B**) Curve of the means endpoints of neutralizing antibodies of CoronaVac vaccinated individuals, (**C**) Linear regression analysis between the neutralization and anti-RBD antibody responses, (**D**) Titer of neutralizing antibodies per day in Dotplot of vaccinated naïve and, (**E**) vaccinated, but SARS-CoV-2 pre-exposed individuals; (**F**) Curve of the means endpoint of neutralizing antibodies of naïve individuals, pre-exposed with negative ELISA anti-RBD prior to vaccination individuals and pre-exposed with positive ELISA anti-RBD prior to vaccination.

We found an increased level of neutralizing antibodies at 2 weeks after the first and second dose with an endpoint titre of 1:20 to 1280 and 1:40 to ≥2560, respectively (Figure 4A,B). Interestingly, the antibodies titres remain high only up to 60 days post vaccination, with a dramatic drop by 120 days. A linear regression analysis showed a strong correlation (r = 0.460, *p* < 0.0001) between the neutralization and anti-RBD antibody responses (Figure 4C). We next decided to dissect the vaccinated groups in those individuals that received only the vaccine (naïve group) and participants with history of pre-exposure to SARS-CoV-2. We found a greater neutralizing response in those vaccinated individuals that have a history of infection prior to vaccination with 100% seroconversion after 1 dose (Figure 4D,E). The naive individuals showed a very different profile with titers up to 1:10 and seroconversion of only 20% after 1 dose, increasing the titers to 1:80 with 77.8% of seropositivity after two doses; however, no neutralizing antibodies were observed after four months in these individuals. Furthermore, in pre-exposed individuals, higher neutralizing antibody levels were observed in those with detectable anti-RBD antibodies in the ELISA prior to immunization (baseline) compared to those who were negative in the anti-RBD ELISA and presented profile very similar to the naïve group with titers up to 1:40 and seropositivity only by 120 days (Figure 4F).

**Figure 5:**
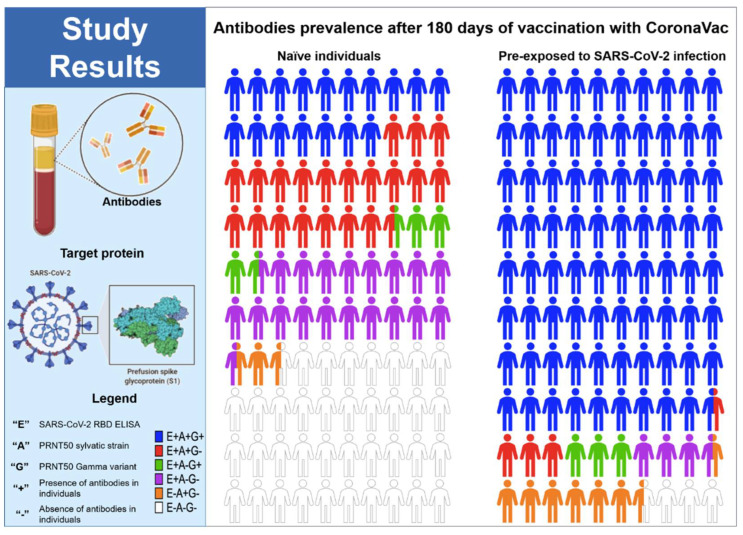
Parts of whole RBD specific and neutralizing antibodies in naïve individuals and individuals pre-exposed to SARS-CoV-2 infection after 180 days of the vaccination with CoronaVac. (+) presence of antibodies in the individuals, (−) absence of antibodies in the individuals, (E) SARS-CoV-2 RBD ELISA, (A) PRNT50 with the B.1.1.33 variant, and (G) PRNT50 with the Gamma variant.

When analysing the action of neutralizing antibodies against the gamma variant, we observed in the naive group a significant decrease in titers and seroconversion (44% after 2 doses) when compared to the results obtained with the primary variant in Brazil (Figure 5) Altogether, these findings indicate the relatively low effect of the CoronaVac vaccine at inducing efficient RBD specific and neutralizing antibody responses in naïve individuals compared to those who were pre-exposed to SARS-CoV-2 infection (Figure 5).

## 4. Discussion

Durability of antibodies against SARS-CoV-2 acquired after natural infection or vaccination is one of the major discussions during this pandemic. Evidence shows that circulating neutralizing antibody levels decrease with time but may remain detectable up to 8–12 months after symptom onset in outpatients and hospitalized patients [16]. However, additional longitudinal studies are needed to elucidate the dynamics and persistence of SARS-CoV-2 specific antibodies in recovered or immunized individuals. It has been reported that individuals who manifest mild COVID-19 disease have a decay of their specific antibodies rapidly during the convalescent phase [17,18,19]. Here, we studied the kinetics of RBD specific antibodies responses, and neutralizing antibodies level in acquired SARS-CoV-2 infection and vaccinated individuals with CoronaVac in the Northern region of Brazil.

In infected people, we observed a maximum seroconversion rate of 88.8%, with peak of RBD responses at 30- and 90-days post infection. This was followed by a gradual decrease after 6 months with 70% seroconversion rate, reaching 11% of seropositivity by 12 months post symptom onset. This data provides solid evidence that humoral immunity against SARS-CoV-2 may not be long-lasting in people with mild disease and is aligned with reports from other countries [17,18,20]. The presence of SARS-CoV-2 specific memory B cells which could be reactivated upon infection has also been noted and would likely contribute to protection from severe disease [21]. It is noteworthy that 14.3% of individuals followed up weekly from the confirmation of infection until 120 days did not seroconvert during this period; this fact has been described in other studies with other populations and is associated with both genetic factors and low viral loads observed in these patients [22].

Overall, in the vaccinated group, a rapid increase in RBD response and neutralizing antibodies levels upon vaccination were observed in individuals with a history of pre-exposure (6–10 months) to SARS-CoV-2 infection compared to naïve individuals and this was independent of gender and age. However, the sharp decline in neutralizing antibodies after 30 days in these pre-exposed individuals demonstrates the need for the booster dose in this period to stimulate the immune system again. Notably, 15 days after booster (2nd dose), we found similar RBD responses between naïve and pre-exposed individuals; this fact has also been reported in those that received CoronaVac (Sinovac) or BNT162b2 (BioNTech/Pfizer, Mainz, Germany) vaccines [16,23,24]. A vaccine cohort with varying immune background (Figure 2D) had anti-RBD antibody profile/responses that appeared similar to the infection-acquired cohort (greater similarity with the naïve population). In those without pre-existing SARS-CoV-2 antibodies from prior infection who were vaccinated with two doses, antibody responses decayed dramatically over the 6-month period, while neutralizing antibodies were observed in these individuals only up to 4 months.

The production of higher neutralizing antibody titers that remained until the end of kinetics (6 months) observed in the pre-exposed individuals were generated exclusively by those who had detectable anti-RBD antibodies in ELISA before vaccination (baseline). Thus, we observed a possible relationship between a robust neutralizing antibody response induced by CoronaVac and the presence of antibodies at detectable levels before vaccination.

We also observed ten individuals with natural infection by SARS-CoV-2 (Gamma variant) after the vaccination with CoronaVac. Nine out of ten did not seroconvert (anti-RBD antibodies) until the moment of the infection; in other words, they presented negative results in the ELISA-RBD after vaccination until the blood draw, which precedes the natural infection by SARS-CoV-2. RT-qPCR confirmed these natural infections. Nine out of ten—but now including the only individual who did not seroconvert—did not have a history of previous infection by SARS-CoV-2 before the vaccination, showing that these naïve individuals may be more susceptible to the infection by SARS-CoV-2 even after the vaccine.

Considering reports showing a tendency for the effectiveness of the COVID-19 vaccines to decrease over time, the WHO and the FDA (Federal Drugs Administration Agency) have recommended a booster dose at six months after the 2nd dose [25]. Vaccination with the mRNA vaccine BNT162b2 (Pfizer–BioNTech) or mRNA-1273 (Moderna, Cambridge, MA, USA) induce higher peak levels of total IgG and neutralising antibodies than seen with CoronaVac, which correlates with a substantial difference in vaccine effectiveness after the second dose [26]. Similar to CoronaVac, the BNT162b2 and mRNA-1273 vaccine antibody responses decline sharply by 6 months; however, the rate of waning differs compared to CoronaVac [27]. At 6 months after the second BNT161b2 mRNA dose, the Spike antibody levels were equivalent to the levels of COVID-19 convalescent individuals [28]. Correspondingly, recent studies have shown a decrease in vaccine effectiveness against SARS-CoV-2 infection of any severity beginning at 3 months after administration of the 2nd dose. For BNT162b2, effectiveness was measured at 92% initially, dropping to 47% at 4–6 months, while for the less efficacious vaccine, ChAdOx1 nCoV-19 effectiveness was 68% initially, but at 4 months onwards no detectable effectiveness was measurable [29]. It will be important to conduct retrospective, cohort studies to determine the protection capacity against hospitalization and severe disease, afforded by 2 doses of CoronaVac over time.

With the rapid evolution of SARS-CoV-2 and the emergence of a new VOC, Omicron, harboring 30 mutations in the spike protein [30], the WHO has recently recommended expanded use of booster doses, with a focus on the most vulnerable people. In accordance with our findings about CoronaVac, the WHO Strategic Advisory Group of Experts on Immunization (SAGE) now recommends expanding booster campaigns, which should be applied between four to six months after the second dose, alongside the application of primary doses [31].

The reduction in the neutralizing potential of antibodies due to the constant appearance of new variants has been a cause for concern. We observed lower neutralizing antibody seropositivity against the Gamma variant (p.1) in naïve individuals, with seroconversion only after the 2nd dose, and a minimum positive rate of 11% after 120 days; in other words, these individuals would be more exposed at around 30% when compared with exposition to the primary strain of SARS-CoV-2 (B.1.1.33) circulating in Brazil.

Given the circulation in Brazil of emerging variants of concern, such as Delta (B1.617.2), which has already been shown in vitro to be 8 times less sensitive for mRNA vaccine-induced antibodies compared to the wild type (WT) [32], and, more recently, Omicron, with preliminary data indicating substantial effect on vaccine efficacy against symptomatic infections and disease [30], there is a need for ongoing assessments of the levels of these antibodies to understand if subsequent infection with this variant in naïve vaccinated individuals restores antibody levels and improves durability, due to the extent of memory responses.

Several countries have recommended different intervals, between 3 and 6 months, based on the epidemiological situation, vaccine availability, and emergence of new variants of concern [11]. To contain the advance of the Omicron variant in Brazil and reduce hospitalizations and deaths, the Ministry of Health also recommended reducing the interval between the second dose and the booster dose of immunization against COVID-19 to four months for all individuals over 18 years of age [11].

The limitation of our study was that we could not evaluate the neutralizing antibodies post-vaccination with Coronavac against the VOC currently circulating and the absence of booster (3rd dose) data in individuals pre-exposed or not exposed to SARS-CoV-2. These data would likely further confirm the trend that prior exposure to SARS-CoV-2 results in enhanced post-vaccination antibody responses, as well as perhaps a third booster would increase antibody levels in naïve individuals to those of pre-exposed individuals. It is worth noting that our results point to the importance of choosing the optimal time to perform the booster dose, and thereby effectively stimulate a robust and more stable antibody response.

In addition to the necessary administration of the timely booster vaccine dose, investigating the feasibility of developing new, updated vaccines based on new variants is crucial to avoid increasing infection rates and preventing the collapse of the health care system.

## 5. Conclusions

Circulating levels of RBD-specific antibodies in those naturally infected with SARS-CoV-2 or vaccinated with two doses of CoronaVac are not durable, falling to undetectable levels after six months. Comparatively, we found that a history of exposure to SARS-CoV-2 with the presence of detectable levels of anti-RBD antibodies in serum in the pre-vaccination period caused superior responses of neutralizing antibodies that were longer lasting. Two doses of the CoronaVac vaccine are not sufficient to induce a high response of neutralizing antibodies, reinforcing the need for a subsequent 3rd booster dose between 4 and6 months after the 2nd dose to prevent infection and symptomatic disease.

## Figures and Tables

**Table 1:** Participant characteristics.

	Variables		Sample Size	Frequency (%)
GP 1—Natural Infection				
Symptomatology	Mild		71	62.3
	Moderate		7	6.1
	Severe		29	25.4
	Asymptomatic		9	6.2
	Gender	M	55	47.4
		F	61	52.6
	Age Group	<50	89 _(44.7 ± 10.9)_	76.7
		≥50	27 _(44.6 ± 11)_	23.3
	**Total**		**116**	
GP 2—Vaccinated				
	With Previous Infection		39	40.6
	Without Previous Infection		57	59.4
	Gender	M	65	67.7
		F	31	32.3
	Age Group ^+^	<50	65 _(44.4 ± 10.8)_	67.7
		≥50	31 _(44.9 ± 11)_	32.3
	**Total**		**96**	

GP—Group; M—Male; F—Female; ^+^—Means; ±—Standard Deviation.

## Data Availability

Not applicable.
